# Two-step feature selection for predicting survival time of patients with metastatic castrate resistant prostate cancer

**DOI:** 10.12688/f1000research.8201.1

**Published:** 2016-11-16

**Authors:** Motoki Shiga

**Affiliations:** 1 Department of Electrical, Electronic and Computer Engineering, Gifu University, Gifu, Japan

**Keywords:** Survival analysis, Cox-proportional hazards model, feature selection

## Abstract

Metastatic castrate resistant prostate cancer (mCRPC) is the major cause of death in prostate cancer patients. Even though some options for treatment of mCRPC have been developed, the most effective therapies remain unclear. Thus finding key patient clinical variables related with mCRPC is an important issue for understanding the disease progression mechanism of mCRPC and clinical decision making for these patients. The Prostate Cancer DREAM Challenge is a crowd-based competition to tackle this essential challenge using new large clinical datasets. This paper proposes an effective procedure for predicting global risks and survival times of these patients, aimed at sub-challenge 1a and 1b of the Prostate Cancer DREAM challenge. The procedure implements a two-step feature selection procedure, which first implements sparse feature selection for numerical clinical variables and statistical hypothesis testing of differences between survival curves caused by categorical clinical variables, and then implements a forward feature selection to narrow the list of informative features. Using Cox’s proportional hazards model with these selected features, this method predicted global risk and survival time of patients using a linear model whose input is a median time computed from the hazard model. The challenge results demonstrated that the proposed procedure outperforms the state of the art model by correctly selecting more informative features on both the global risk prediction and the survival time prediction.

## Introduction

Prostate cancer is the most common malignant tumor among men and ranks third in terms of mortality after lung cancer and colorectal cancer. The major clinical treatment against prostate cancer is an anti-androgen therapy to inhibit male hormones providing to prostate cancer cells. However, the therapy cannot inhibit the cancer cell growth for long because these cells can develop the resistance against the androgen absence condition. This developed prostate cancer is called metastatic castrate resistant prostate cancer (mCRPC), which is the major cause of death in prostate cancer patients ^1, 2^. Even though some options for treatment of mCRPC have been developed, the most effective therapies remain unclear ^3^. Finding key clinical variables related with mCRPC is an important first step for understanding the disease progression mechanism and clinical decision making for these patients. Halabi *et al.* ^4^ identified key factors of mCRPC from a lot of clinical variables by feature selection based on a Cox’s proportional hazards model with a *L*_1_ penalty, *i.e.* a variant of Lasso for survival analysis ^6, 7^ and built a mCRPC prognostic model. This data-driven approach is important to correctly predict patient health status for treatment choices. To validate and improve such prediction models of mCRPC patients, larger scale clinical datasets collected from several clinical institutes are useful. The Prostate Cancer DREAM challenge in DREAM 9.5 ( https://www.synapse.org/ProstateCancerChallenge) provided such datasets and an opportunity to tackle this essential challenge using the wisdom of the crowd, in which participating teams were required to submit prediction models based on clinical variables from the comparator arms of four phase III clinical trials with over 2,000 mCRPC patients treated with first-line docetaxel. My method for this challenge consists of a two-step feature selection procedure, which first performs both sparse feature selection ^7^ and statistical hypothesis testing ^8^, and then performs a forward feature selection ^9^ to screen out non-informative features. Selected clinical variables were used to build a prognostic model to predict global risks of patients. For a survival time prediction, my method further used a linear model fitting with median survival time ^5^ computed by the established progression model. The final result of this DREAM challenge demonstrated that, in the sub-challenge 1a, the proposed procedure outperforms Halabi’s model ^4^ by correctly selecting more informative features on global risk prediction. In sub-challenge 1b, my method using these selected features predicted the survival time more correctly and outperforms most of the other team’s methods.

## Methods

### Dataset and pre-process

Data across comparator arms of four phase III clinical trials have been compiled, annotated, cleaned and were made available through the Challenge and remain available on the web site ^7^. These datasets include over 150 clinical variables and over 2,000 mCRPC patients treated with first-line docetaxel. The output value to be predicted for unknown new patients is the survival time. The survival times of patients are not always observed because some patients are still alive when they are lost to follow-up or when the study ends. Thus the observed survival times are right censoring. For the training dataset, three of the clinical trial cohorts were provided, which includes data for 476, 598, and 526 patients from clinical trial ASCENT-2 (Novacea, provided by Memorial Sloan Kettering Cancer Center) ^10^, VENICE (Sanofi) ^11^, and MAINSAIL (Celgene) ^12^, respectively. For the test dataset, 470 patients’ data were provided from clinical trial ENTHUSE-33 (AstraZeneca) ^13^. The goal of this challenge was to correctly predict global risk of death and survival time of patients in the test dataset. In these datasets, clinical variables for some patients were missing. These missing values were imputed by the median of each variable for numerical values and by the most frequent value for each categorical variable.

### Hazard model

A Cox proportional hazards model is assumed for the relationship between clinical variables (input variables) of a patient and the survival time (a output variable) ^5^. Let ***x*** be clinical variables of a patient. The hazard function of the patient at time *t* is given by

$$h(t|x)=h_{0}(t)\;\exp(\beta^{T}x),$$

where *h*_0_( *t*) is a baseline hazard function and ***β*** is a weight vector to be optimized from training data. When the weight value of the *d*-th clinical variable *β* ***_d_*** is large, the clinical variable is informative to predict the survival time. On the other hand, when *β* ***_d_*** =0, the *d*-th clinical variable is independent with the survival time. Thus the correctly estimating ***β*** is the most important task in survival analysis. A common estimation is performed by maximizing a partial log likelihood function of *N* patients given by

$$L(\beta )=\sum_{n=1}^{N}\delta_{n}\left[\beta^{T}x_{n}-\log\{\sum_{j\in R_{n}}\exp\left(\beta^{T}x_{j}\right)\}\right],$$

where ***x _n_*** is a vector of clinical variables of the *n*-th patient, *δ _n_* is a binary variable. *δ _n_* = 1 for died patients and *δ _n_* = 0 for right-censored patients at time *t _n_* when is the survival time of the *n*-th patient. *R _n_* is the risk set at time *t _n_*. This estimation is of course affected by non-informative clinical variables (noise variables) because the size of the training data is limited, where the number of clinical variable is large but the number of patients is small. Before estimating weight vector ***β*** in the hazard function, my method implemented a two-step feature selection to screen out non-informative clinical variables.

### Feature selection

The goal of feature selection is to divide the set of all clinical variables into a set of informative variables and non-informative variables by optimizing the final scoring metric. However, this optimization is NP-hard, *i.e.* intractable in general. Thus my procedure implemented this task in a heuristic manner; 1) screening numerical features by a *L*_1_ sparse penalized regression and categorical features by a statistical hypothesis testing, and then 2) a forward sequential feature selection to narrow the list of informative selected features by optimizing the final scoring metric. For the first procedure, my procedure used a variant of LASSO for a Cox’s proportional hazards model ^7^ provided by R package glmpath ^11^. This approach should choose the weight of the *L*_1_ penalty term. My method automatically chose it by minimizing an information criterion (AIC), which is a criterion to estimate the generalized error. Because the computational cost of this implementation with a lot of clinical variables is expensive, my procedure used this sparse feature selection for only numerical variables to reduce the computational cost. Categorical variables were evaluated using rank statistical hypothesis testing ^5, 8^. This method tests if there is a significant difference between two or more survival curves with different values of a categorical variable. If the difference of curves is statistically significant, the categorical variable might be related with survival times of patients. Therefore, such variables should be selected for a survival time prediction model.

Among selected features described above, my procedure further implemented a forward feature selection ^9^ to narrow the list of clinical variables. In my procedure, the most useful feature that maximally increases an integrated time-dependent AUC (iAUC) ^14^, which is the final scoring metric in sub-challenge 1a, is sequentially added one by one until all variables are selected. After that, the optimal set of clinical variables is selected by maximizing iAUC. iAUCs were estimated by cross-validation (CV), which was performed by randomly splitting all training data into 90% training data and 10% test data. iAUC was estimated as the median among ten calculated iAUC values.

### Prediction of global risk of death and survival time

After selecting informative features, parameter ***β*** in the Cox proportional hazard function was optimized using only the selected clinical variables. Next, the hazard function was used to predict the global risk of death for each patient ^5^. The survival time of each patient can be predicted based on the median time when an estimated survival probability is equal to 0.5, computed from the hazard function ^5^. However the root mean squared error of this prediction method was still large and an estimation bias was included because of the right censoring setting, which will be experimentally demonstrated later. Against this problem, my method used a linear model fitting from computed median times to observed survival times in the training dataset. Survival time was predicted by the liner regression model whose input is the estimated median time of each patient.

## Results

### Selected clinical variables

**Figure 1.**
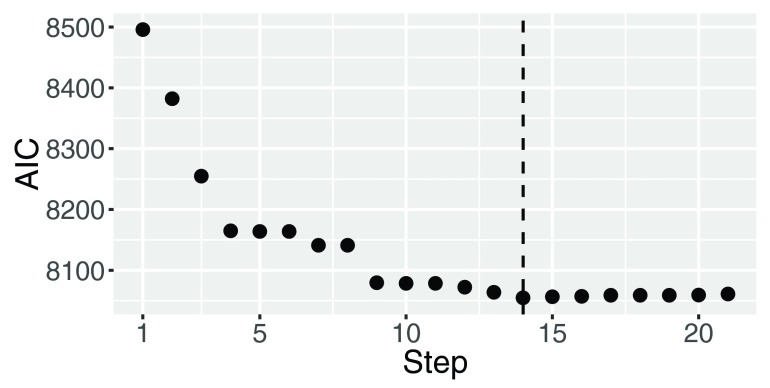
AICs of steps in the *L*_1_ regularization path.

My method removed clinical variables having a lot of missing values and then it used only 14 numerical clinical variables and 56 categorical clinical variables with less number of missing values. Feature selection for numerical clinical variables was first implemented using the *L*_1_ penalized approach ^7^ by function coxpath in R package glmpath ( https://cran.r-project.org/web/packages/glmpath/glmpath.pdf). This function can compute the entire regularization path for the *L*_1_ penalized model by increasing the weight of the penalty and check only steps of the path when a weight parameter of a clinical variable becomes greater than zero. Table 1 shows the first 20 steps and the sequence of added clinical variables. Figure 1 shows computed AIC scores of these steps. The best feature set (step) was selected by minimizing an AIC score. This procedure chose the 14th step and then selected nine clinical covariates (ENTRTPC, ALP, HB, AST, ECOGC, NEU, PLT, PSA and LDH) as informative clinical variables.

**Table 1.** Selected clinical variables at each step of the regularization path.

Step	Clinical variable
1	ENTRT_PC
2	ALP
3	HB
6	AST
8	ECOG_C
11	NEU
12	PLT
13	PSA
14	LDH
15	CA
16	CREAT
18	ALT
19	WBC
20	TBILI

On the other hand, differences of survival curves by categorical clinical variables were statistically tested using function survdiff in R package survival ( https://cran.r-project.org/web/packages/survival/survival.pdf). Table 2 shows the ranking result of clinical variables with *p*-values. The threshold of a significance level was set to 0.05 and then the procedure selected categorical features ANALGESICS, MHGEN, MI, TURP, MHCARD, ACE_INHIBITORS, MHPSYCH and PROSTATECTOMY.

**Table 2.** *p*-value of statistical hypothesis testing for categorical clinical variables.

Rank	Clinical variable	*p*-value
1	ANALGESICS	9.8e-08
2	MHGEN	8.5e-03
3	MI	1.0e-02
4	TURP	1.2e-02
5	MHCARD	1.3e-02
6	ACE_INHIBITORS	2.6e-02
7	MHPSYCH	3.9e-02
8	PROSTATECTOMY	4.3e-02

**Figure 2.**
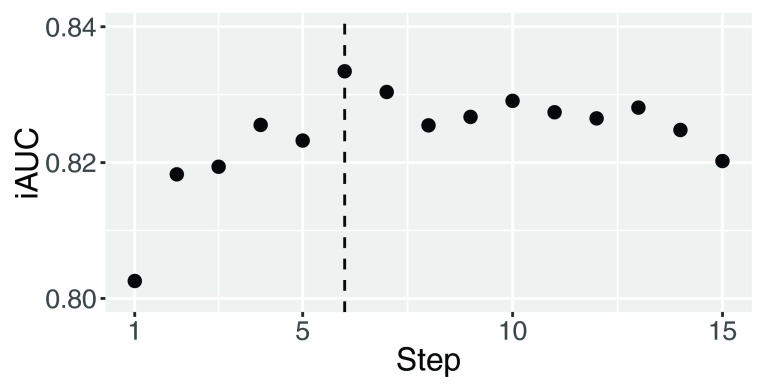
iAUC at each step of the forward feature selection.

For these 17 selected clinical variables by two feature selections, we further implemented the forward feature selection described in the previous section. Figure 2 shows iAUC at each step of the forward feature selection. This figure shows that the step maximizing AUC is the sixth step which includes six clinical variables ALP, AST, ECOG_C, HB, MI and PLT. These clinical variables were finally selected to predict global risks and survival times of patients.

## Prediction performance

The parameter vector ***β*** of a Cox-proportional hazards model with six selected clinical variables was optimized by maximizing the partial log-likelihood function. Then the global risks of death of patients in the test dataset were predicted from the optimized model. Prediction performance iAUC by the proposed method is 0.7671 although iAUC by Halabi’s model is 0.7429, which can be found in the ranking result of sub-challenge 1a in the web site of Prostate Cancer DREAM Challenge ( https://www.synapse.org/ProstateCancerChallenge). This result demonstrated that the proposed prediction outperforms Halabi’s method by correctly selecting informative features.

**Figure 3.**
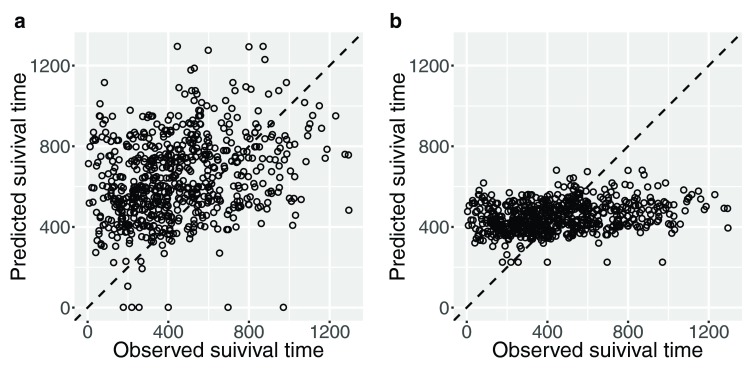
Predicted survival times in the training dataset.

Furthermore, survival times of patients were predicted using median times computed from the optimized hazard model. Figure 3(a) shows predicted values and observed values in the training dataset. This result demonstrates that the estimation of variance is large and the center of plotted data is located to the upper-left from the diagonal line, meaning that predicted values are biased. To improve these prediction errors, the median survival times were transformed by a linear model. Figure 3(b) shows the prediction result after this transformation. These figures demonstrate that the proposed prediction reduces both the estimation bias and variance. As a result, the root mean square error (RMSE) between true values and predictions is drastically improved, from 281.3 by median survival times to 198.7 by the proposed method. This prediction result in sub-challenge 1-b in the Prostate Cancer DREAM Challenge was ranked in the group of top-performers even though the global risk prediction result in sub-challenge 1a was worse than the best 10 performers.

## Conclusions

This paper outlines a prediction method of global risks of mCRPC patients for sub-challenge 1a and that of survival time for sub-challenge 1b in the Prostate Cancer DREAM Challenge. The challenge result in sub-challenge 1b demonstrated that this procedure, which is based on the two-step feature selection and the correction of naïve survival time predictions from the optimized hazard model, outperformed the other teams’ methods. Especially, for survival time prediction, this correction method based on centering and reducing estimation variance works well to improve RMSE, the scoring metric of sub-challenge 1b. This analysis demonstrates that a naïve prediction from a basic model (Cox’s proportional hazards model) is not always optimal for an evaluation metric. Thus a suitable transformation is necessary to optimize the metric.

This paper also provides a two-step feature selection procedure because using only a single feature selection method leaves a lot of non-informative features. By carefully selecting features by this two-step procedure, the global risk prediction outperformed Halabi’s model ^4^ in sub-challenge 1a. This result demonstrated that multiple feature selection procedures are necessary to screen out non-informative features. Future work includes the validation of informative clinical variables selected by not only of the method proposed here, but also other top-performing methods. Table 3 shows the comparison of our selected clinical variables with Halabi’s selected variables ^4^. Both models selected ALP, ECOG_C and HB but neither our model nor Halabi’s model selected the other eight clinical variables. Although selection results depend on the datasets used, we should further investigate the importance of these clinical variables using knowledge in clinical and biological research areas.

**Table 3.** Selected clinical variables by the proposed model and Halabi’s model ^4^.

Clinical Variables	Proposed Model	Halabi’s Model
ALB	×	○
ALP	○	○
ANALGESICS	×	○
AST	○	×
ECOG_C	○	○
HB	○	○
LDH	×	○
LIVER	×	○
MI	○	×
PLT	○	×
PSA	×	○

## Data availability

The data referenced by this article are under copyright with the following copyright statement: Copyright: © 2016 Shiga M

Data associated with the article are available under the terms of the Creative Commons Zero "No rights reserved" data waiver (CC0 1.0 Public domain dedication). 

The Challenge datasets can be accessed at: https://www.projectdatasphere.org/projectdatasphere/html/pcdc

Challenge documentation, including the detailed description of the Challenge design, overall results, scoring scripts, and the clinical trials data dictionary can be found at: https://www.synapse.org/ProstateCancerChallenge

The code and documentation underlying the method presented in this paper can be found at: http://dx.doi.org/10.7303/syn4229266 ^15^
