# Supplementary material for: Two-step feature selection for predicting survival time of patients with metastatic castrate resistant prostate cancer
Source: F1000Res. 2016 Nov 16;5:2678. [Version 1] doi: 10.12688/f1000research.8201.1 (PMC5130083; doi:10.12688/f1000research.8201.1)
Supplement: Peer review history

# Peer review: Referee response for version 1

**DOI:** 10.5256/f1000research.8821.r17685

**Published:** 2016-11-28

**Authors:** Ka Yee Yeung

**Affiliations:** 1 Institute of Technology, University of Washington, Tacoma, WA, USA

This paper is generally well-written, with a clear and concise description of the problem and challenge. The author adopted a two-step feature selection procedure: a penalized *L*_1_ regression for Cox PH model (R package "glmpath") in the first step, and forward selection in the second step. Features are selected to optimize the iAUC (integrated time-dependent AUC) in 10-fold cross validation.

**Major comments:**

 I am confused about how the two-step feature selection procedure works. The author mentioned the following

- " *Among selected features described above, my procedure further implemented a forward feature selection to narrow the list of clinical variables*." 
- " *This figure shows that the step maximizing AUC is the sixth step which includes six clinical variables ALP, AST, ECOG_C, HB, MI and PLT. These clinical variables were finally selected to predict global risks and survival times of patients*."

Therefore, I assume the second step starts with the features selected from the first step. However, the features shown in Table 2 don't appear to be a subset of the features shown in Table 1. Also, the feature "MI" doesn't appear to be in Table 1.

**Minor comments:**

- The difference between sub-challenge 1a and sub-challenge 1b is not documented in the Introduction. Please explain that in sub-challenge 1a, the submissions consist of the risk scores, while in sub-challenge 1b, the submissions consist of the predicted survival time. 
- Under Results and "Selected clinical variables", the author mentioned that " *My method removed clinical variables having a lot of missing values and then it used only 14 numerical clinical variables and 56 categorical clinical variables with less number of missing values*.".  What are the exact criteria for filtering clinical variables given that there are 150+ clinical variables to start with? 
- Please explain what the clinical variables mean (e.g. ENTRTPC, ALP, HB, AST, ECOGC, NEU, PLT, PSA and LDH in Table 1).
- Please expand the captions for Table 1 and Table 2 to put these tables in the context of the 2-step feature selection procedure.
- In Table 3, I assume the circle means "yes" and the cross means "no".  Please add a legend to the caption.

I have read this submission. I believe that I have an appropriate level of expertise to confirm that it is of an acceptable scientific standard.

# Peer review: Referee response for version 1

**DOI:** 10.5256/f1000research.8821.r17683

**Published:** 2016-11-25

**Authors:** Niels Richard Hansen, Søren Wengel Mogensen

**Affiliations:** 1 Department of Mathematical Sciences, University of Copenhagen, Copenhagen, Denmark

This paper offers methods to calculate patient risk scores and predict survival times from proportional hazard models in the context of the Prostate Cancer DREAM Challenge. The author used a two-step feature selection procedure by first using a combination of the LASSO and significance testing and then using a forward selection method.

**The challenge consisted of two parts. **In one part the contestants were to assign global risk scores to patients and in the other they were to predict survival times. The author states that the results of the methods in question for the former outcome did not make it into the top-10 of the challenge. However, the paper seems to conclude that the two-step feature selection is superior to one-step feature selection. This is possibly based on a comparison with the DREAM benchmark model only. In this case, the paper would benefit from a more specific statement.

 For the feature selection it seems unclear if the LASSO variable selection was done conditionally on the categorical predictors (without penalizing their coefficients) or marginally on only the continuous predictors.

**Cross-validation** seems to have been carried out incorrectly in the sense that only the second step (the forward selection) and not the first step was cross-validated. Whether this has consequences for the quality of the selection is unclear, but the estimated iAUC-values reported in Figure 2 are suspiciously large – and they definitely overestimate the validation iAUC.

**For predicting survival times**, the author first used a fitted proportional hazards model to estimate median survival times. Then observed survival times were regressed linearly on the predicted medians. This estimated a linear transformation, which could be used to transform predicted medians to means. The paper would benefit from a brief discussion of the motivation behind this approach. It is stated that the linear transformation “reduces both the estimation bias and variance”, which is unclear as it is not stated what we’re aiming to estimate. Arguably, estimating the means from the medians should improve the performance as the RMSE is used to score the predictions.

**Minor comments:**

 p. 5: 1-b→1b

 Table 3: Please add information to the caption about what the symbols mean. It is clear from reading the paper that “open circle” means “selected”, but that is not self-evident.

We have read this submission. We believe that we have an appropriate level of expertise to confirm that it is of an acceptable scientific standard.
